# Role of self-criticism in postpartum mental health: a network analysis

**DOI:** 10.1186/s41155-024-00321-2

**Published:** 2024-09-14

**Authors:** Bruna Cardoso Gerhardt, Jovana Giacobo Serra, Camila Zimmer, Adriane Xavier Arteche

**Affiliations:** Pontifícia Universidade Católica do Rio Grande do Sul, Post Graduate Program in Psychology, Porto Alegre (RS), Brazil

**Keywords:** Self-criticism, Self-compassion, Postpartum, Pandemic, Network analysis

## Abstract

**Purpose:**

A significant percentage of women experience psychopathological symptoms during the postpartum period, which can impact not only their mental health and well-being but also the relationship between mother and baby. However, studies investigating how specific psychological factors, such as self-compassion and self-criticism, contribute to the development and maintenance of these symptoms are scarce.

**Methods:**

This cross-sectional study aimed to examine the relationship among compassionate self-responding (CSR), uncompassionate self-responding (USR), maternal mental health indicators, mother-infant bonding, and the perceived impact of the COVID-19 pandemic. Data were collected online from 189 Brazilian women with infants aged 0 to 12 months. Participants completed measures of sociodemographic characteristics, Self-Compassion Scale (SCS), Edinburgh Postnatal Depression Scale (EPDS), Postpartum Specific Anxiety Scale (PSAS-BR-RSF-C), Postpartum Bonding Questionnaire (PBQ), and COVID-19–Impact on Quality of Life (COV19-QoL).

**Results:**

Through network analysis, our findings highlighted that postpartum depression played a central role in the structuring of variables in this system. Furthermore, USR, instead of CSR, emerged as the variable most strongly associated with levels of postpartum depression, which, in turn, was associated with mother-infant bonding.

**Conclusion:**

Mitigating levels of self-criticism in mothers may represent a pathway to prevent the development of postpartum depression, which, in turn, could impact the quality of the mother-infant relationship. Given the significance of the child’s early years for their emotional development, intervening early in maternal mental health may be a means to prevent mental disorders in the child as well.

## Introduction

The transition to motherhood entails significant intra and interpersonal changes in a woman's life, often resulting in negative emotional outcomes, such as the onset or exacerbation of mental disorders (Cantwell, 2021). Throughout the postpartum period, symptoms of depression and anxiety are commonly observed, affecting a substantial portion of this population (Chen et al., 2022). These conditions not only affect maternal well-being but also bear implications for the mother-infant relationship (Fallon et al., 2021; Rocha et al., 2020). Studies have demonstrated that children whose mothers experience postpartum depression are at a significantly higher risk of developing cognitive, emotional, social, and behavioral difficulties (Aoyagi et al., 2019; Murray & Cooper, 1996; Slomian et al., 2019). Therefore, there is a critical need for research to address maternal mental health and explore mother-infant bonding, with the aim of identifying potential intervention targets to enhance the overall postpartum experience.

The COVID-19 pandemic has further intensified the mental health concerns faced by postpartum mothers. Studies have documented heightened prevalence rates of depression and anxiety symptoms during this period, underscoring the necessity of investigating the pandemic's impact on postpartum mental health. In their systematic review and meta-analysis, Gao et al. (2022) examined 20,225 postpartum women across 16 countries spanning four continents (Asia, Europe, North America, and South America) during the COVID-19 pandemic. The study revealed a prevalence of 26.7% for depression symptoms and 33.8% for anxiety symptoms among mothers of newborn children. In Brazil, Loret de Mola et al. (2021) found that out of 1042 participants, 29.3% were classified as probable cases of postpartum depression based on the Edinburgh Postnatal Depression Scale (EPDS; Cox et al., 1987; Santos et al., 2007). This research, part of a cohort study, revealed that the prevalence of depressive symptoms in this population was 5.7 times higher during the pandemic compared to data obtained before COVID-19.

The presence of psychopathological symptoms can be detrimental to mother-infant bonding. A systematic review conducted by Slomian et al. (2019) analyzed 122 studies on the untreated consequences of postpartum depression. The results revealed that mothers experiencing depression encountered more substantial challenges in their relationships with their children during the first year of life. In terms of breastfeeding, there was a negative correlation between postpartum depression and breastfeeding practices, which could result in an earlier cessation of exclusive breastfeeding. Moreover, mothers with postpartum depression reported greater dissatisfaction with their breastfeeding experience. Regarding maternal care, the review found a significant link between postpartum depression and increased difficulties in caring for the baby, with depressed mothers exhibiting poorer parenting practices compared to non-depressed mothers.

In efforts to prevent and alleviate psychopathological symptoms while promoting mental health, the concept of self-compassion has received extensive attention in recent decades. Self-compassion is defined as a healthy way of relating to oneself, characterized by a welcoming, warm, and non-evaluative stance towards one’s own suffering (Neff, 2003). The most widely used method for evaluating this construct is the Self-Compassion Scale (SCS; Neff, 2003), which is designed to assess self-compassion through a total factor. It incorporates scores from self-compassion subscales (self-kindness, common humanity, and mindfulness) as well as the inverse scores from self-criticism subscales (self-judgement, isolation, and over-identification). Based on this, research has identified associations between self-compassion and various outcomes, including negative correlations with depression (MacBeth & Gumley, 2012) and anxiety (Neff et al., 2007), and positive associations with well-being (Zessin et al., 2015), quality of life (Mantelou & Karakasidou, 2017), and emotional regulation (Inwood & Ferrari, 2018).

In the context of motherhood, preliminary studies have shown that self-compassion is linked to fewer symptoms of depression and anxiety (Felder et al., 2016; Fonseca & Canavarro, 2018), as well as to engagement in more well-being-related behaviors (Simpson et al., 2021), increased psychological flexibility, and stronger mother-infant bonding (Whittingham & Mitchell, 2021). Moreover, experimental studies have indicated that interventions targeting self-compassion lead to increased maternal self-compassion and significant reductions in depressive symptoms (Fonseca et al., 2019; Guo et al., 2020) and negative affect (Angus et al., 2024). In the context of the COVID-19 pandemic, Fernandes et al. (2021) investigated maternal self-compassion and the mother-infant bond, finding that maternal self-compassion appears to play a crucial role in stabilizing the mother-infant bond during the postpartum period, particularly in the context of the pandemic.

According to Neff (2003), self-criticism is the opposite of self-compassion and is characterized by self-judgment and negative self-evaluation in response to perceived failure or suffering. Neff posits that self-compassion and self-criticism lie on a bipolar continuum, which is why the self-critical components are included in the total factor provided by the SCS. However, recent literature advocates for considering these constructs as independent components (Halamová et al., 2021; Kumlander et al., 2018; López et al., 2015; Muris & Otgaar, 2020), which can be labeled as compassionate self-responding (CSR) and uncompassionate self-responding (USR) (Brenner et al., 2017; Ferrari et al., 2022; Muris et al., 2021). This recommendation arises from the observation that inverse scores for USR may inflate the total factor of self-compassion, and because self-criticism has been associated with higher rates of psychopathology compared to the positive components of self-compassion (Mills et al., 2007; Muris & Petrocchi, 2017). Furthermore, the use of the two factors allows for more accurately identifying which components are associated with the variables being researched.

When considering the perinatal period, a study evaluated the effect of an experiment on self-compassion using the six subscales of the SCS. The results indicated that the mechanisms of change were primarily due to a reduction in USR items rather than an increase in CSR items (Angus et al., 2024). In the same direction, initial evidence suggests that self-criticism serves as a vulnerability factor for the development of postpartum depression (Pedro et al., 2019). Furthermore, another study revealed that mothers exhibiting higher levels of self-criticism demonstrated poorer attachment quality with their children at 20 months of age (Gravener et al., 2012). Thus, it is inferred that excessive self-criticism may influence the bond between mother and infant, thereby influencing the emotional development of the child. However, literature on this topic remains limited, hindering the formation of a consensus regarding the role of self-criticism in maternal mental health.

Given the established predictive role of self-criticism in various mental disorders (Auerbach et al., 2014; Cohen et al., 2013; Fennig et al., 2008; Pedro et al., 2019; Werner et al., 2019), and the lack of studies evaluating this concept in mothers, there is a critical need to investigate this construct and its impact on maternal mental health. Such research is crucial for preventing mental disorders not only in mothers but also in their infants. With these considerations in mind, the present study aims to contribute to the existing national and international literature by examining potential relationships among self-compassion, self-criticism, indicators of maternal mental health, mother-infant bonding, and the perceived impact of the COVID-19 pandemic on quality of life.

## Method

### Procedures

This research comprises a cross-sectional, quantitative, and descriptive study conducted via the online platform Qualtrics Survey Software (https://www.qualtrics.com/pt-br/). A convenience sample of Brazilian mothers was recruited through social media platforms from March to November 2022. The inclusion criteria for the study were (1) Brazilian nationality; (2) age 18 years or older; (3) having at least one child between 15 days and 12 months old. Exclusion criteria included (1) incomplete questionnaire responses; (2) a baby with a severe illness or syndrome, as reported by the participant in response to the question "Was your baby born with any severe illness or syndrome?” in the sociodemographic questionnaire. Initially, 262 individuals began filling out the survey. Participants with incomplete questionnaire responses (*n* = 71) or those reporting babies affected by severe illnesses or syndromes (*n* = 2) were excluded from the sample, resulting in a final sample of 189 participants. Upon completion of the study, participants received a booklet containing psychoeducational information on mental health in motherhood, prepared by the researchers involved in this study (Gerhardt: Maternal self-compassion in the context of the COVID-19 pandemic: Associations with depression, anxiety, and quality of the mother-baby relationship, unpublished). All variables, including diagnoses, were assessed using self-reported questionnaires.

### Ethical procedures

#### Ethical procedures

This study was approved by the Research Ethics Committee (CEP) of the Pontifical Catholic University of Rio Grande do Sul (PUCRS) on February 4^th^, 2022, under CAAE number: 55069122.0000.5336. The ethical aspects proposed by the Guidelines and Standards provided for in Resolution No. 510 of the National Health Council (2016) and Resolution 466 of 2012 (CNS 466/2012) were respected. All participants accepted the Informed Consent Form (TCLE) before starting to fill in the instruments.

### Materials

#### Sociodemographic Questionnaire

Maternal-related demographic questions and infant characteristics were asked at the beginning of the survey (see Table 1 for means and frequencies). The questionnaire collected information regarding the women’s age, marital status, occupational situation, level of education, family income, parity, history of psychopathology, gestational diabetes (yes vs. no), type of delivery time (vaginal vs. c-section), baby's gender, time since delivery (baby’s age), and information related to COVID-19.

**Table 1:** Maternal and infant characteristics (*n*=189)

**Variable**	**Level**	**Counts**	**%**
Marital Status	Single	28	14.8
	Married	103	54.5
	Co-habiting	56	29.6
	Divorced	2	1.1
In a relationship	Yes	177	94.1
	No	11	5.9
Educational Attainment	Incomplete Elementary Education	1	0.5
	Elementary Education	1	0.5
	Incomplete High School Education	2	1.1
	High School Education	18	9.5
	Technical Education	6	3.2
	Incomplete Higher Education	30	15.9
	Complete Higher Education	48	25.4
	Postgraduate	83	43.9
Occupation	Student	12	6.3
	Employed	76	40.2
	Unemployed	29	15.3
	Beneficiary	1	0.5
	Self-employed	60	31.7
	Other	11	5.8
Family income	Less than R$2.000	26	13.8
	Between R$2.000 and R$6.000	69	36.5
	Between R$6.000 and R$9.000	28	14.8
	Up to R$9.000	66	34.9
Diagnosis of psychopathology	Yes	101	53.4
	*Depression*	31	31
	*Anxiety*	38	38
	*Depression and anxiety*	12	12
	*Others *	19	19
	No	88	46.6
Birth order	1st	132	69.8
	2nd	45	23.8
	3rd and after	12	6.3
Type of delivery	Vaginal	88	46.6
	Cesarian	101	53.4
Baby's sex	Female	83	44.1
	Male	105	55.9

#### Validated psychological measures

COVID-19–Impact on Quality of Life (COV19-QoL; Repišti et al., 2020): A unidimensional scale developed with the objective of measuring the impact of the COVID-19 pandemic in the quality of life. Participants rate their experiences over the past seven days by responding to six questions (e.g., “I think my quality of life is lower than before”) on a 5-point Likert scale ranging from 1 (completely disagree) to 5 (completely agree). The total score is obtained by averaging the scores across all items, with higher scores indicating a greater perceived impact of the pandemic on quality of life. The scale demonstrated good internal consistency, with Cronbach’s alpha exceeding 0.80 for both populations. For the present study, the scale was translated into Brazilian Portuguese and subjected to psychometric analyses, including confirmatory factor analysis, which yielded good fit indices (CFI = 1.000; TLI = 1.012; RMSEA = 0.000) and reliability indices (Cronbach’s *α* = 0.87; McDonald’s *ω* = 0.87).

Self-Compassion Scale (SCS; Neff, 2003; translated and adapted by de Souza & Hutz, 2016). The scale consists of 26 items on a 5-point Likert scale. It includes six subscales that measure the CSR components (self-kindness, common humanity, and mindfulness) and USR components (self-judgment, isolation, and overidentification). The measurement method used was the bi-factor model, generating reliability indices for each subscale: CSR (McDonald’s *ω* = 0.92) and USR (McDonald’s* ω* = 0.91). The internal consistency level of the scale’s validation and adaptation for Brazil, using the total factor, was *α* = 0.92 (de Souza & Hutz, 2016).

Edinburgh Postnatal Depression Scale (EPDS; Cox et al., 1987; validated for the Brazilian population by Santos et al., 2007). It consists of a self-assessment scale comprising 10 items referring to depressive symptoms in the postpartum period, perceived in the last seven days. The total score is based on the sum of the items. The study of the Brazilian version indicates the cutoff point ≥ 10 as adequate for indicative of postpartum depression. The scale has good internal consistency indexes (Cronbach’s *α* = 0.87). In the present study, the instrument, measured from its total score, maintained a good reliability index (McDonald’s *ω* = 0.89).

Postpartum Specific Anxiety Scale–Research Short-form–for Global Crises (PSAS-BR-RSF-C; Silverio et al., 2021; validated for the Brazilian population by Gerhardt et al. (Gerhardt et al.: Psychometric properties of the Postpartum Specific Anxiety Scale research short-form for use during global crises in a Brazilian population (PSAS-BR-RSF-C), in preparation)). A 12-item self-report scale was designed to evaluate the individual's level of postpartum anxiety. The factorial structure of the scale includes four factors: maternal competence anxiety and attachment, anxiety for the baby’s safety and well-being, anxiety about practical baby care, and psychosocial adaptation to motherhood. The total score is calculated by summing the item scores, with a cutoff point of 26 indicating a significant level of anxiety. The original scale demonstrated good reliability (McDonald’s *ω* = 0.87). The instrument was translated into Brazilian Portuguese by Araújo et al. (2024) and its psychometric properties were evaluated prior to this study, showing a reliability index of *ω* = 0.77 for the Brazilian population.

Postpartum Bonding Questionnaire (PBQ; Brockington et al., 2006; translated and adapted into Brazilian Portuguese by Baldisserotto et al., 2018, psychometrically validated by Baldisserotto et al., 2022). The questionnaire consists of 12 items, reduced from the original 25 items during the validation process. Participants respond on a scale of 0 (never) to 5 (always), with higher scores indicating difficulties in mother-infant bonding. The Brazilian validated version includes three factors: loss of interest, rejection and anger, and anxiety and risk of abuse. A second-order factor analysis revealed a general Impaired Bonding factor. The reliability of the three dimensions was good (*α* = 0.83, 0.79, and 0.69), as well as the overall scale (McDonald’s *ω* = 0.83) used in the present study.

### Data analysis

Data were stored at Statistical Package for the Social Sciences (SPSS) (version 28), and all statistical analyses were conducted using Jeffreys’s Amazing Statistics Program (JASP; version 0.15). Descriptive analyses were conducted to examine the characteristics of the participants, including sociodemographic factors, levels of self-compassion and self-criticism, indicators of mental health difficulties (postpartum depression and postpartum anxiety), mother-infant bonding, and perceived impact of the COVID-19 pandemic on quality of life. The Shapiro–Wilk test was used to assess the distribution of variables, with *p* ≤ 0.05 indicating a non-normal distribution. Only respondents providing full data were included in the analysis. Participants with incomplete data had not answered two or more instruments; therefore, imputation methods were not applied.

Spearman correlation analyses were performed to explore the relationships between the variables. Reference values for interpreting the correlations were applied, considering weak (0.1 to 0.29), moderate (0.3 to 0.49), and strong (values greater than 0.5) correlations (Cohen, 1988). Subsequently, a network analysis was conducted using the Graphical Least Absolute Shrinkage and Selection Operator algorithm (GLASSO; Friedman et al., 2008). This approach allows for the examination of partial correlations, which reveal the associations between variables while controlling for the effects of other variables. This helps to provide a more accurate understanding of the data, avoiding spurious correlations. The resulting graph organizes the variables based on the strength of their associations, with variables having higher centrality indicating more associations (Machado et al., 2015). Additionally, network analysis provides descriptive measures such as centrality indices, which can be utilized to model or predict network processes, including the flow of information through a variable (Costantini et al. 2014). While there are various centrality measures, four commonly used indices include: (1) *betweenness*, this index indicates the extent to which a node facilitates the flow of information between other nodes in the network; (2) *closeness*, reflecting the proximity of a variable to others, this index measures the inverse of the sum of the shortest path distances from the node to all others; (3) *strength*, representing the cumulative weight of paths connecting a node to others, this index signifies the overall influence or importance of the node in the network, and (4) *expected influence*, this index assesses the extent to which a node is expected to influence others based on its connectivity patterns.

## Results

### Participants

The sample of this study comprised 189 Brazilian women, aged between 18 and 44 years (*M* = 31.4; SD = 5.5). Among the participants, 92 resided in Rio Grande do Sul (48.6%), 32 in São Paulo (16.9%), and 9 in Paraná (6.9%), while the remaining participants were distributed across other Brazilian states. The average age of the babies at the time of data collection was 5.19 months (SD = 3.89). Regarding social support, 64% of the mothers indicated having a good support network (*n* = 121), and 75.1% believed that the pandemic had not affected the number of people who were assisting them during this period (*n* = 142). Additionally, 60.3% of the sample (*n* = 114) scored above the cutoff for postpartum depression on the EPDS (≥ 10), while 53.9% (*n* = 102) scored above the cutoff for postpartum anxiety on the PSAS-BR-RSF-C (≥ 26).

### Correlations between sociodemographic and outcome variables

Table 2 shows bivariate Spearman correlations between all study variables. Maternal age demonstrated a significant and moderate association with depression *(r* = − 0.34; *p* < 0.001), and a weak association with anxiety *(r* = − 0.28; *p* < 0.001) and USR *(r* = − 0.29;* p* < 0.001), indicating that older mothers exhibited lower levels of depressive and anxious symptoms as well as reduced self-criticism. Family income exhibited a negative and moderate association with EPDS *(r* = − 0.30;* p* < 0.001), suggesting that higher income levels were associated with decreased depression. Individuals with a diagnosis of psychopathology displayed a significant and weak association with depression *(r* = − 0.28; *p* < 0.001) and mother-infant bonding *(r* = − 0.26;* p* < 0.001), and a moderate correlation with USR *(r* = 0.35; *p* < *0.0*01) and CSR *(r* = − 0.30;* p* < 0.001). The perception of social support was weakly linked to depression *(r* = 0.25;* p* < 0.001) and USR *(r* = 0.23;* p* < 0.001), with a positive perception (e.g., “I believe that I have a good support network”) correlating with reduced depression and self-criticism.

**Table 2:** Spearman correlations between demographic and outcome variables

	1	2	3	4	5	6	7	8	9	10	11
1. Mother’s age	—										
2. Education	0.56**	—									
3. Family income	0.52**	0.48**	—								
4. Psychopathology	0.07	0.10	0.06	—							
5. Social support	0.00	0.00	− 0.13	− 0.03	—						
6. PSAS-BR	− 0.28**	− 0.12	− 0.18*	− 0.17*	0.14	—					
7. EPDS	− 0.34**	− 0.19*	− 0.30**	− 0.28**	0.25**	0.61**	—				
8. COV19-QoL	− 0.18*	− 0.03	− 0.07	− 0.15	0.12	0.36**	0.55***	—			
9. USR	− 0.29*	− 0.11	− 0.21*	− 0.35**	0.23**	0.52**	0.71***	0.46***	—		
10. CSR	0.17*	0.07	0.13	0.30**	− 0.14	− 0.26**	− 0.48***	− 0.27**	− 0.61**	—	
11. PBQ-12	− 0.16	− 0.02	− 0.08	− 0.26**	0.16	0.44**	0.55**	0.27**	0.42**	− 0.17*	—

*Note:* *p < 0.01, **p < 0.001. *In bold* moderate to strong correlation, *PSAS-BR* Postpartum Specific Anxiety Scale–Short Version for Global Crises, *EPDS* Edinburgh Postnatal Depression Scale, *COV19-QoL* COVID-19 – Impact on Quality of Life, *USR* Uncompassionate Self-Responding, *CSR* Compassionate Self-Responding, *PBQ-12* Postpartum Bonding Questionnaire

Significant associations were observed among all the instruments used in this study. In terms of CSR, negative and weak associations were identified with postpartum anxiety *(r* = − 0.26; *p* < 0.001) and the impact of the pandemic on quality of life *(r* = − 0.27; *p* < 0.001). Furthermore, negative and moderate associations were found with depression *(r* = − 0.48; *p* < 0.001), while impairments in the mother-infant exhibit weak correlations with CSR *(r* = − 0.17;* p* = 0.014). As for USR, significant associations were found with all variables, featuring moderate correlations with the perceived impact of the pandemic *(r* = 0.46; *p* < 0.001) and mother-infant bonding *(r* = − 0.42;* p* < 0.001), and strong correlations with postpartum depression *(r* = − 0.71; *p* < 0.005) and anxiety *(r* = − 0.52;* p* < 0.001).

### Network analysis

**Fig. 1:**
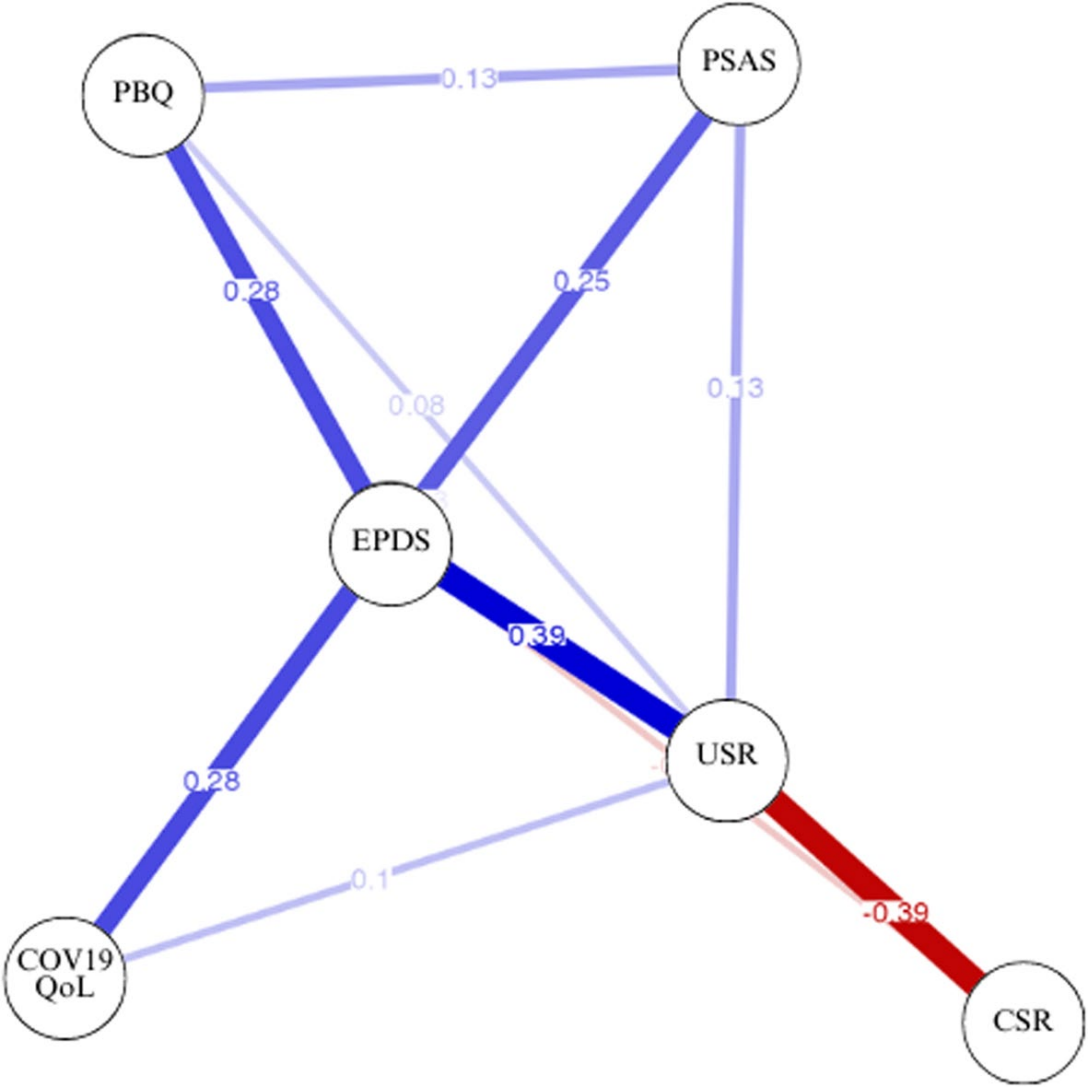
Network analysis (CSR and USR). *Note:* Abbreviations: COV19-QoL = COVID-19–Impact on Quality of Life; EPDS = Edinburgh Postnatal Depression Scale; PSAS-BR = Postpartum Specific Anxiety Scale–Short Version for Global Crises; PBQ_12 = Postpartum Bonding Questionnaire; USR = Uncompassionate Self-Responding; CSR = Compassionate Self-Responding. Figure provided by JASP (version 0.15)

**Fig. 2:**
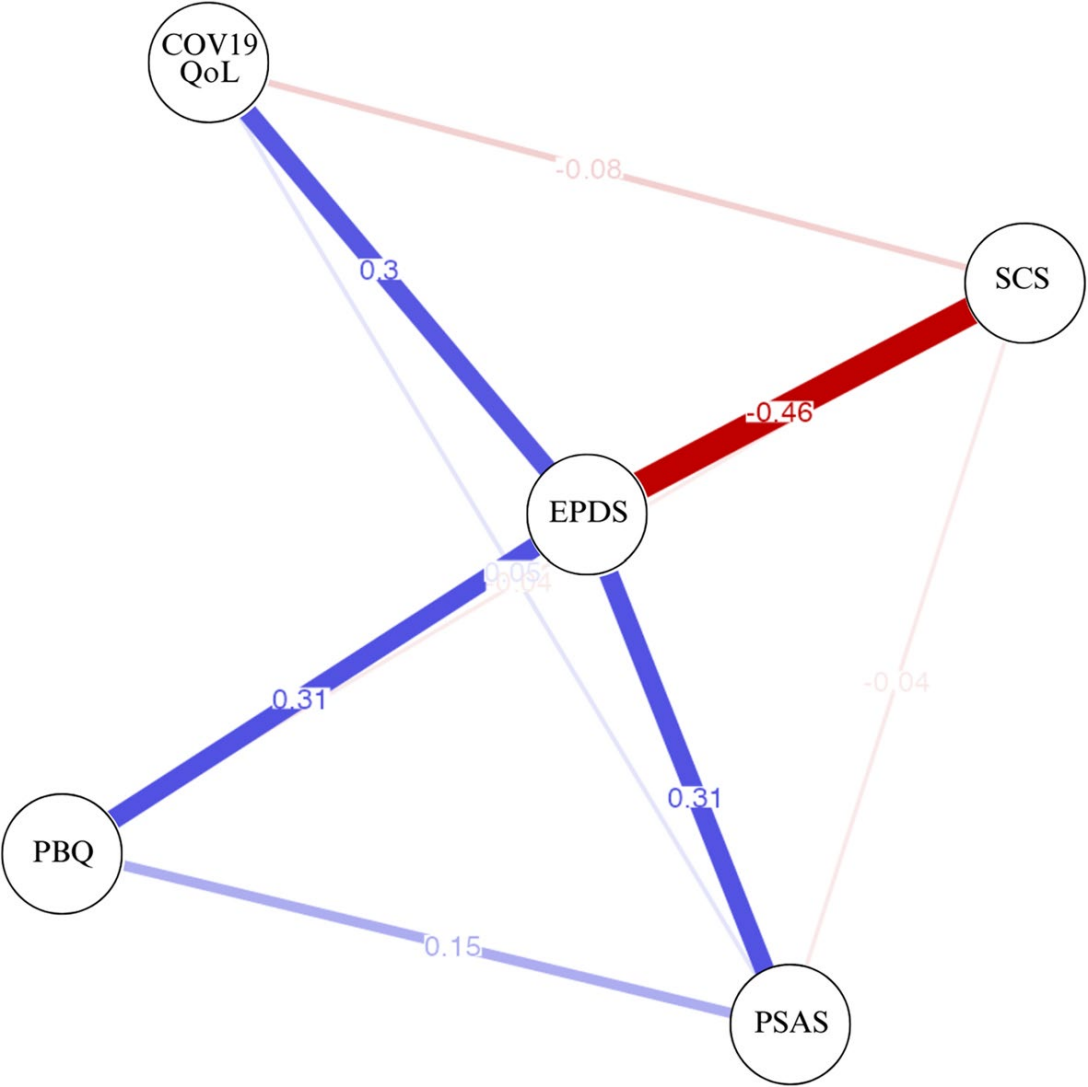
Network analysis (Self-compassion Scale Total Score). *Note:* Abbreviations: COV19-QoL = COVID-19–Impact on Quality of Life; EPDS = Edinburgh Postnatal Depression Scale; PSAS-BR = Postpartum Specific Anxiety Scale–Short Version for Global Crises; PBQ_12 = Postpartum Bonding Questionnaire; SCS = Self-compassion Scale Total Score. Figure provided by JASP (version 0.15)

In the network analysis, sociodemographic variables such as mother’s age, family income, perception of social support, and diagnosis of psychopathology formed a separate cluster and were independent of the main system. Therefore, these variables were excluded from further analysis. The final model (Fig. 1) revealed that postpartum depression played a central role in the organization of the model, directly linked with postpartum anxiety, impairment in the mother-infant bond, USR, and the impact of the pandemic on quality of life. Impairment in the mother-infant bond was primarily associated with postpartum depression, indicating its dependence on these symptoms. Surprisingly, impaired mother-infant bonding showed no significant relationship with anxiety levels or the perceived impact of the pandemic. Additionally, CSR did not emerge as a significant factor, except for its association with USR. An alternative model (Fig. 2) utilizing the total self-compassion score was conducted, and it exhibited a significant relationship with the rest of the system. However, when considering the separate components of CSR and USR, the association between CSR and the other variables primarily occurred through USR rather than CSR itself.

Regarding centrality measures, as shown in Table 3, EPDS demonstrates the highest levels of betweenness, closeness, strength, and expected influence, underscoring its substantial influence within the system. Similarly, USR garners a moderately high strength centrality score, indicating its significant importance in the network. Overall, depression and self-criticism emerge as the most influential variables based on their scores, playing pivotal roles in facilitating connections and exerting influence within the network. In contrast, anxiety, CSR, the impact of COVID-19 on quality of life, and mother-infant bonding exhibited lower centrality scores, suggesting that they wield less influence within the network.

**Table 3:** Centrality measures per variable

	Network			
Variable	Betweenness	Closeness	Strength	Expected influence
EPDS	1.84	1.77	1.54	1.43
PSAS-BR	− 0.58	− 0.65	− 0.46	0.26
USR	0.49	0.63	0.98	-0.16
CSR	− 0.58	− 0.65	− 0.62	-1.68
COV19-QoL	− 0.58	− 0.55	− 0.78	0.02
PBQ-12	− 0.58	− 0.54	− 0.65	0.11

*Note* *:* *EPDS* Edinburgh Postnatal Depression Scale, *PSAS-BR* Postpartum Specific Anxiety Scale–Short Version for Global Crises, *COV19-QoL* COVID-19–Impact on Quality of Life, *PBQ-12* Postpartum Bonding Questionnaire

## Discussion

The aim of this study was to investigate the association between self-compassion and self-criticism with maternal mental health outcomes and mother-infant bonding. A cross-sectional design was used and we conducted a network analysis on data obtained from Brazilian mothers. Our findings indicate that self-criticism is strongly and significantly associated with maternal mental health, highlighting its importance in developing potential interventions in this area. Moreover, our results emphasize the importance of regarding compassionate self-responding and uncompassionate self-responding as separate constructs rather than diametrically opposed traits. These findings reinforce existing literature that advocates for incorporating the bi-factor model of the SCS into research and practice (Brenner et al., 2017; Ferrari et al., 2022; Halamová et al., 2021; Muris & Otgaar, 2020).

The reported diagnosis of a psychopathological condition, along with the scores on the EPDS and PSAS-BR-RSF, are crucial factors to consider as they may have influenced the outcomes of this study. The prevalence rates observed in this study are higher than those reported in other Brazilian studies that investigated postpartum depression symptoms during the pandemic, which reported rates of 29.3% of probable cases based on the EPDS (Loret de Mola et al., 2021), as well as international studies that identified a prevalence of 26.7% for depression symptoms and 33.8% for anxiety symptoms (Gao et al., 2022). One explanation for these findings is rooted in the recruitment methodology utilized in this study, which involved the use of social media alongside an image captioned “Participate in research on the mental health of postpartum women.” It is hypothesized that women already receiving mental health care may have been more inclined to participate in this study. In relation to the lack of association between diagnoses and the scores of EPDS and PSAS-BR-RSF-C, one hypothesis is that the questionnaires evaluate current active symptoms, whereas the reported diagnosis pertains to lifelong conditions.

While CSR demonstrated significant correlations with all examined variables, the network analysis model using partial correlations did not support these findings. Specifically, CSR was only associated with USR, suggesting that this was its primary mode of influence. This outcome aligns with recent research advocating for the differentiation of these two constructs (Costa et al., 2016; Halamová et al., 2021; Kumlander et al., 2018; Muris & Otgaar, 2020; Muris & Petrocchi, 2017). Literature suggests that the items assessing self-criticism, which are inversely scored in the total self-compassion score provided by the SCS, may artificially inflate its overall measurement. In the current study, upon exploring the network analysis using the total score and the two separate components, it became evident that CSR lacked significant associations with levels of depression and anxiety unless linked to USR. Conversely, USR exhibited the strongest and most significant correlations with these psychopathologies. Based on these findings, our study concurs with the authors who advocate for measuring CSR and USR, rather than the total factor of the SCS. Additionally, other scales such as the Forms of Self-Criticising and Self-Reassuring Scale (FSCRS; Baião et al., 2015) can be utilized to measure both concepts separately.

One hypothesis for the results concerning CSR is rooted in the perception that self-compassion may be culturally associated with various myths, such as indulgence, weakness, self-pity, complacency, and selfishness (Neff, 2015). Additionally, societal expectations and idealizations of the maternal role may contribute to the amplification of self-criticism when mothers perceive a discrepancy between societal demands and their self-perception (Arrais et al., 2014; Douglas & Michaels, 2005). This misalignment can significantly impact the development and persistence of psychopathological symptoms, necessitating interventions that extend beyond adopting a self-kind posture. Excessive self-criticism may possess deeply ingrained characteristics that cannot be effectively addressed through self-compassion alone. The study aligns with the suggestion made by Halamová et al. (2021) that addressing excessive self-criticism may require adopting an assertive and protective stance.

Network analysis provides valuable insights into potential intervention targets within a system, with the results of this analysis offering significant implications. Notably, the graphical representation of node disposition suggests a potential predictive relationship between USR and levels of depression, which is associated with levels of anxiety and the perceived impact of the COVID-19 pandemic on quality of life. Additionally, corroborating existing literature, depression appears to predict impairments in mother-infant bonding. Considering centrality measures, interventions targeting self-criticism and postpartum depression are crucial for influencing other variables within the network. These findings underscore the interconnectedness of these variables and emphasize the potential efficacy of interventions targeting self-criticism in reducing postpartum depression levels, which could subsequently alleviate impairments in mother-infant bonding.

The low scores related to anxiety symptoms have led to controversial hypotheses. The lack of methodological consensus (Matthey et al., 2003; Pawluski et al., 2017; Silverio et al., 2021), symptoms concealed by measures targeting postpartum depression (Loyal et al., 2020), and discrepancies regarding prevalence (Dennis et al., 2017) suggest a possible underreporting of maternal anxiety cases, as well as greater difficulty in understanding the disorder and identifying its impacts (Dennis et al., 2017; Goodman et al., 2016). In this study, an innovative scale was employed to specifically measure postpartum anxiety (Gerhardt et al.: Psychometric properties of the Postpartum Specific Anxiety Scale research short-form for use during global crises in a Brazilian population (PSAS-BR-RSF-C), in preparation; Silverio et al., 2021), and the results indicate that these symptoms are associated with higher levels of depressive symptoms but do not have a direct impact on the quality of the mother-infant relationship. It is hypothesized that depression symptoms can lead mothers to experience greater difficulties in connecting with their babies, as well as having reduced energy to attend to their needs (Kornfield et al., 2021; Righetti-Veltema et al., 2002). Conversely, maternal anxiety may result in increased concerns regarding the baby and lead to overprotection (Jones et al., 2021). Although the literature highlights the negative impacts of overprotection over time (Van Der Bruggen et al., 2008; Van Petegem et al., 2022), in the context of mother-infant bonding during the first year, it may not necessarily pose an issue. Studies with broader and more representative samples, utilizing specific measures for the assessment of postpartum anxiety, are necessary to initiate the development of a consensus in the literature about this topic.

Although these results suggest potential intervention pathways, it is important to consider the limitations of this study. The sample size and diversity may not allow for generalization to the entire Brazilian population. The majority of participants were residents of Rio Grande do Sul and São Paulo, primarily from urban and well-developed areas. Additionally, the sample predominantly comprised women with higher education levels, which could restrict the generalizability of the findings. Furthermore, data collection was conducted online, and all measures were self-reported, introducing the possibility of uncontrollable biases. The self-reported nature of the data might also be susceptible to social desirability bias, potentially affecting the accuracy of the reported information.

## Conclusion

The network analysis conducted in this study offers valuable insights for guiding future interventions. We propose conducting randomized controlled trials (RCTs) to assess the effectiveness of intervention programs aimed at mitigating postpartum depression, with a specific focus on reducing self-criticism and fostering self-compassion strategies. Our findings underscore the importance of addressing cultural misconceptions surrounding self-compassion and cultivating a protective and assertive approach to counteract excessive self-criticism. For instance, interventions like the Emotion-Focused Training for Self-Compassion and Self-Protection (EFT-SCP; Halamová et al., 2021) show promise in this regard. Furthermore, we recommend integrating psychoeducation, psychological flexibility, emotion regulation abilities, and guidance on enhancing social support and parenting skills into these interventions. Moreover, longitudinal studies are highly recommended to assess the sustained impact of these interventions on postpartum depression and mother-infant bonding. Following participants over an extended period will allow for the evaluation of long-term benefits on maternal mental health and child well-being, thus advancing our understanding of effective strategies in this critical area.

## Data Availability

The data that support the findings of this study are available from the corresponding author upon reasonable request.

## Abbreviations

EPDS: Edinburgh Postnatal Depression Scale
SCS: Self-Compassion Scale
CSR: Compassionate Self-Responding
USR: Uncompassionate Self-Responding
PSAS-BR-RSF-C: Postpartum Specific Anxiety Scale–Research Short-form–for Global Crises
PBQ: Postpartum Bonding Questionnaire
COV19-QoL: COVID-19 Impact on Quality of Life
CFI: Comparative Fit Index
TLI: Tucker Lewis Index
RMSEA: Root mean squared error of approximation
